# Conditions that influence the impact of malpractice litigation risk on physicians’ behavior regarding patient safety

**DOI:** 10.1186/1472-6963-14-38

**Published:** 2014-01-25

**Authors:** Erik Renkema, Manda Broekhuis, Kees Ahaus

**Affiliations:** 1 University of Groningen, Faculty of Economics and Business, Operations Department, P.O. Box 800, 9700, AV Groningen, The Netherlands

**Keywords:** Physicians, Malpractice litigation risk, Patient safety

## Abstract

**Background:**

Practicing safe behavior regarding patients is an intrinsic part of a physician’s ethical and professional standards. Despite this, physicians practice behaviors that run counter to patient safety, including practicing defensive medicine, failing to report incidents, and hesitating to disclose incidents to patients. Physicians’ risk of malpractice litigation seems to be a relevant factor affecting these behaviors. The objective of this study was to identify conditions that influence the relationship between malpractice litigation risk and physicians’ behaviors.

**Methods:**

We carried out an exploratory field study, consisting of 22 in-depth interviews with stakeholders in the malpractice litigation process: five physicians, two hospital board members, five patient safety staff members from hospitals, three representatives from governmental healthcare bodies, three healthcare law specialists, two managing directors from insurance companies, one representative from a patient organization, and one representative from a physician organization. We analyzed the comments of the participants to find conditions that influence the relationship by developing codes and themes using a grounded approach.

**Results:**

We identified four factors that could affect the relationship between malpractice litigation risk and physicians’ behaviors that run counter to patient safety: complexity of care, discussing incidents with colleagues, personalized responsibility, and hospitals’ response to physicians following incidents.

**Conclusion:**

In complex care settings procedures should be put in place for how incidents will be discussed, reported and disclosed. The lack of such procedures can lead to the shift and off-loading of responsibilities, and the failure to report and disclose incidents. Hospital managers and healthcare professionals should take these implications of complexity into account, to create a supportive and blame-free environment. Physicians need to know that they can rely on the hospital management after reporting an incident. To create realistic care expectations, patients and the general public also need to be better informed about the complexity and risks of providing health care.

## Background

Despite ongoing efforts during the last 10 years to increase patient safety in hospitals, the number of adverse events has not been reduced, nor have hospitals become safer [1,2]. A prerequisite for patient safety is that physicians behave in the interest of their patients. Although practicing safe behavior is an intrinsic part of a physician’s ethical and professional standards, physicians practice behaviors that run counter to patient safety. These behaviors include practicing defensive medicine [3,4], failing to report incidents [5-7], and hesitating to disclose incidents to patients [8,9]. Defensive medicine includes performing unnecessary medical procedures [10] and tests [11], deviating from guideline practices [12] and avoiding high-risk patients [3]. Malpractice litigation risk influences physicians’ behaviors that run counter to patient safety [3,6-11]. After an incident, patients may start legal action against health care workers to prevent similar incidents in the future, to find out how the incident happened and why, to receive financial compensation, or to hold staff or organizations accountable for their actions [13,14]. In this paper, we identify influences on the relationship between the risk of malpractice litigation and the unsafe behaviors of physicians.

Prior research has offered many explanations for these patient unsafe behaviors. For example, physicians may practice defensive medicine to preserve the doctor-patient relationship [15]. Most harm occurs when incidents are not reported [16]. A lack of time, a scarcity of feedback on previously reported incidents, a rejection of bureaucracy, and associating reporting more with nursing discourage physicians from reporting incidents [5,16,17]. Physicians may also fail to disclose incidents to patients because of the health services’ blame culture [18], a lack of confidence in their communication skills [9], a lack of insight into patients’ and relatives’ experience and understanding of incidents, and the challenge of dealing with patients’ and colleagues’ emotions [19].

According to several studies, the risk of malpractice litigation leads to the practice of defensive medicine [3,4,10-12]. Worries about the financial burden and high cost of liability insurance premiums also seem to be positively linked to practicing defensive medicine [3]. A recent review study identified the fear of legal consequences and disciplinary actions to be an important barrier for reporting incidents [6]. Moreover, litigation risk discourages physicians from disclosing incidents to patients [8,9]. Healthcare workers may fail to provide patients with all requested information after an error, because of perceived inadequacies of legal protections from disclosure laws [20]. If physicians believe that disclosure makes patients less likely to sue, they are more likely to strongly endorse disclosure [21].

Additional factors may affect the relationship between physicians’ malpractice litigation risk and behaviors that run counter to patient safety. Our aim is to identify these additional conditions, which may increase our understanding of the underlying mechanisms of this relationship. This could help healthcare managers and professionals to create a supportive and blame-free environment, which reduces defensive medicine and encourages physicians to report incidents and disclose them to patients. We identified these conditions using an exploratory field study consisting of 22 in-depth interviews with stakeholders in the litigation process.

## Methods

Qualitative interviews are a useful method for uncovering the meanings and understandings of the informants. They give an opportunity to explore how informants describe experiences and practices that are the object of research [22]. From November 2010 until April 2011, we conducted qualitative interviews to explore the conditional factors that affect the impact of the risk of malpractice litigation on behaviors that run counter to patient safety. To ascertain all potentially relevant factors, we consulted the potentially accused (physicians and hospitals) and other relevant stakeholders in the litigation process. None of the researchers occupies roles in any of the organizations that participate in the litigation process, or have a role in the litigation process itself. We focused on organizations that are involved in all stages of the litigation process. We included 20 organizations: ten hospitals, three governmental healthcare bodies, three law institutions, two insurance companies, one physician organization, and one patient organization. Members of these organizations are in direct contact with physicians during different stages in the litigation process. We approached 22 members of these organizations in The Netherlands for a semi-structured interview, and obtained written informed consent from all study participants. We interviewed five physicians, two hospital board members, five patient safety staff members from hospitals, three representatives from governmental healthcare bodies, three healthcare law specialists, two managing directors from insurance companies, one representative from a patient organization, and one representative from a physician organization. The in-depth interviews of 1 to 2 hours followed a broad thematic guide (see the 'Key areas of interview guide' section), and were aimed at gathering narratives about contextual factors that can influence the relationship between malpractice litigation risk and unsafe behaviors. The interview notes were analyzed using the qualitative analysis program NVivo (version 9). Two team members (ER and MB) engaged in topic and analytical coding. However, to ensure rigor, all three authors contributed to the analysis through meetings where they discussed the codes. Thus, we operationally developed the codes and themes using an iterative process that followed a “grounded” approach [23]. After having analyzed interviews with participants from all stakeholders in the litigation process, further data collection was considered not to yield any new insights and was terminated. We identified four conditions that influence the relationship, which we explore in the following section with example quotations to illustrate our findings and interpretation.

### Key areas of interview guide

Handling of complaints and malpractice litigation

Impact of malpractice litigation on physicians

Reporting of incidents and the role of litigation

Open communication about incidents within hospitals and towards patients

Occurrence of defensive medicine

Relevant factors for physicians to deal with litigation

Responsibility for incidents

## Results

### Conditional factors that affect the relationship between malpractice litigation risk and physicians’ behavior

The first conditional factor that was revealed by our analysis was the complexity of care given. Complexity of care refers to care with multiple and or interwoven problems and interventions [24]. One respondent referred to the difference in public opinion about routine-based errors and errors that arise from complex care:

“Most errors are routine-based and are due to a loss of concentration or dedication. These errors are difficult to discuss with the patient. It is a public opinion that routine-based errors are not acceptable. On the contrary, very specialized care provides more space to make errors. An error during a difficult surgical operation is more accepted than a child who dies during birth while the only thing the gynecologist could answer is 'we were constantly checking the heart rate monitor'.” (Hospital board member, also a physician)

If errors from complex care are more accepted by the public, complexity of care might act as a mitigating factor on the relationship between the risk of malpractice litigation and the failure of physicians to report and disclose incidents to patients, or directly encourage these behaviors.

Another respondent mentioned a different effect:

“For some complex surgeries, patients have to go abroad because surgeons do not want to carry out these operations due to the fear of being litigated in case of an adverse outcome.” (Managing director of an insurance company)

This quote indicates that for certain complex cases, litigation fear can result in defensive medicine to such an extent that physicians refuse to treat a patient. Then, complexity does not mitigate the relationship between malpractice litigation risk and defensive medicine, but has the opposite effect. It strengthens this relationship or has a direct increasing effect on defensive medicine.

The second conditional factor that we identified is whether physicians discuss incidents with colleagues:

“How partnerships react to incidents, complaints, and claims differs and is of great importance because the opinion of other doctors is important for the individual physician to reflect: did I do it right? Some partnerships discuss errors or near accidents openly, and others do not. Within our partnership of gynecologists, a colleague is asked to support a physician when he is called to the disciplinary court. A complaint leads to practicing more defensively and can have a life-long impact.” (Hospital board member, also a physician)

“The process (of disciplinary litigation) is painful and could take up to 2 years. In our hospital, four physicians, with disciplinary litigation experience, have formed a help group to assist colleagues who are confronted with the Disciplinary Committee.” (Patient safety staff, hospital)

These quotes illustrate that discussing adverse events with colleagues is important for a physician and can give support when coping with the risk of litigation. Therefore, discussing incidents with colleagues can act as a mitigating factor on the relationship between physicians’ malpractice litigation risk and behaviors that run counter to patient safety, or can have a direct effect on these behaviors.

A third influence on the relationship between malpractice litigation risk and physician behavior is personalized responsibility. According to literature from the field of organizational behavior and psychology, personalized responsibility implies that responsibility for a task belongs exclusively to an individual rather than being dispersed, shared, or undefined [25]. In practice, responsibilities are often dispersed:

“Feeling more responsible than one’s actual responsibility is most common. But, with complex patients treated by different physicians, it sometimes happens that a physician does not feel the responsibility to act because he has a different view of the treatment than the physician in charge.” (Patient safety staff, hospital)

Responsibilities also can be off-loaded:

“If something goes wrong the responsibility is often unloaded on colleagues.” (Representative of a patient group)

Personalized responsibility touches the core of the medical profession and is, according to some of the patient safety staff members we spoke to, too sensitive to discuss within the hospital. Next to being factual responsible, physicians generally have a strong feeling of responsibility towards their patients, but these quotes suggest this sense of responsibility varies. When something goes wrong physicians might even shift the blame towards colleagues, as mentioned in the second quote. In that case it’s also likely to presume that physicians then fail to report and disclose incidents to patients. One of our respondents mentioned a process that is being used to encourage the reporting of incidents:

“SURPASS is an instrument used in the surgical process to call for a ‘time-out’ during an operation. Reflection moments should be built-in when working in teams. If anything goes wrong, this can be discussed together and one can decide who on behalf of the team is going to report the adverse event; to prevent that reporting is experienced as betrayal.” (Representative of a patient group)

The last factor addressed by our respondents was the response of the organization to its physicians after an incident:

“The hospitals mostly refuse physicians, nurses, or other personnel to appear in a court case as a witness, if called by the public prosecutor. They want to protect their personnel.” (Representative of a governmental body)

“The quality department of this hospital protects physicians against the ‘outside world’, if physicians report a calamity themselves, show regret, and cooperate fully in the process. Only if the physician has behaved recklessly will the hospital not continue to assist the physician and provide a lawyer for the physician.” (Patient safety staff, hospital)

Little is known about the differences between the ‘internal’ organization’s response to incidents and the ‘external’ response of litigation or disciplinary actions through the legal system. The last statement illustrates an attempt by the hospital’s quality-control department to reduce the impact of legal punishment on its physicians, in the case that thoughtful and responsible behavior nevertheless caused a calamity. The respondent mentioned that physicians consult the quality department for guidance when there has been an incident, for information about the practicalities and to find out what they should report. By responding in a non-punitive way, hospitals create a safe environment for incident reporting. This could reduce the impact of malpractice litigation risk on physicians’ behavior, and thereby reduce defensive medicine behavior and increase the willingness to report incidents and to disclose incidents to patients.

## Discussion

Our exploratory field study revealed four conditional factors that influence the impact of the risk of malpractice litigation on physicians’ behaviors that run counter to patient safety. How these factors influence this relationship will be discussed in this section.

If care for a patient becomes more complex, then the risk of an adverse event occurring increases [26]. Complex care also involves higher mortality rates, which increases calls for accountability [27]. Because of these aspects, complex care might increase the impact of litigation risk on physician behavior and lead to more defensive medicine. A study in which physicians stated that they avoid high-risk patients in response to the fear of malpractice litigation supports this hypothesis [3]. At the same time, if the public considers complex errors more acceptable, physicians may be encouraged to report or disclose incidents to patients. The exact effect of this condition requires further study.

The discussion of mistakes among colleagues or team members is an important element of ‘team psychological safety’ (i.e., the shared belief that it is safe to take interpersonal risks within a team). This leads to a climate of safety and supportiveness that enables people to ‘embrace’ error and to seek feedback [28]. The literature reports that discussing medical errors with colleagues helps physicians to adapt their future behavior to prevent medical errors [29]. If discussing adverse events with colleagues has similar effects on their perceived malpractice litigation risk, then it might encourage incident reporting and disclosure of incidents to patients. This could be related to the complexity of care. Research shows that complexity is positively linked to the amount of information exchanged between health care team members [24]. Therefore, there could be a relationship between complexity and discussing incidents with colleagues, which might have a joint effect on the relationship between litigation risk and physician behavior.

Personalized responsibility, in contrast to team responsibility, makes an individual more identifiable and increases the expectation of having to justify one’s action or decision [30]. Therefore, personalized responsibility is expected to increase the impact which malpractice litigation risk has on physicians’ behavior that run counter to patient safety. Personalized responsibility is also linked to complexity. Research shows that delivering complex services often involves more and different kinds of health care professionals (see, for example, [24,31]), which makes it harder to pinpoint responsibilities [32]. This way complexity of care interacts with personalized responsibility and further research is needed to understand their joint effect on the relationship between litigation risk and physicians' behavior.

We make a distinction between an internal response to incidents by the hospital organization and an external response by the legal system. Legal systems in most countries hold physicians individually accountable for errors. In some countries, the implementation of incident-reporting systems is accompanied by regulations that oblige the hospital management to create an atmosphere in which physicians do not have to fear that incident reporting will lead to punitive measures. According to some researchers, a non-punitive response to errors leads to an increase in the number of adverse events reported [33]. Therefore, the organization’s response could also directly affect the physicians’ behaviors.

The effects of the conditional factors that we have identified may differ for different forms of behavior. Complex care could encourage reporting and disclosure if its risk is more accepted by the public, but at the same time, it could increase defensive medicine because of the impact of litigation risk. The conditions that were identified in this study do not have a direct effect on patients’ intentions to sue. However, their effect on the disclosure of incidents may indirectly affect patients’ intentions to sue, because one reason for litigation is to find out how and why things happened.

There are several limitations to our field study. The number of physicians was limited. A study using a larger group of physicians might uncover additional forms of behavior that are affected by the risk of malpractice litigation, and conditional factors that may affect the relationship between physicians’ malpractice litigation risk and behavior. We conducted this study in one country, The Netherlands. Malpractice litigation differs amongst countries. In the United States, the UK, The Netherlands and many other Western countries, medical malpractice claims are processed in a tort system. Patients need to prove that a physician was negligent and show that negligence caused their suffering. Some countries, such as New Zealand and the Nordic countries of Europe, use an administrative compensation system in which physicians are not exposed to financial litigation. The behavior of physicians in response to the risk of malpractice litigation may differ depending on how much physicians are exposed to the risk of litigation, and how they perceive this risk. We did not study the impact of the risk of malpractice litigation on other behavioral responses, e.g. physicians’ compliance to patient safety recommendations such as the use of checklists during surgery. A systematic cross-industry review did not report the risk of malpractice litigation as a factor for noncompliance with safety rules [34].

Despite these limitations, this study raises some important issues. From previous research it’s known that care complexity increases the need to share information between health care professionals that are involved in the provision of care. In these settings, procedures should be put in place for how incidents will be discussed, reported and disclosed. The lack of such procedures can lead to the shift and off-loading of responsibilities, and the failure to report and disclose incidents. Hospital managers and healthcare professionals should take these implications of complexity into account, to create a supportive and blame-free environment. Physicians need to know that they can rely on the hospital management after reporting an incident. This awareness could stimulate the willingness to report incidents and to disclose them to patients. To create realistic care expectations, patients and the general public also need to be better informed about the complexity and risks of providing health care.

## Conclusion

In the Hippocratic oath, physicians promise to behave ethically and professionally to their patients. However, the risk of malpractice litigation makes it very hard for physicians to apply this behavior. Our study showed four conditional factors that could help physicians to apply this behavior and reduce the impact of malpractice litigation risk: complexity of care, discussing incidents with colleagues, personalized responsibility, and hospitals’ response to physicians following incidents. Besides, research indicates that other methods than litigation are probably more fruitful in stimulating health care professionals to comply with safety standards. The implementation of a just culture, a focus on communication and learning from errors, and working collaboratively in teams founded on trust and mutual respect [35] may be more promising routes to create safer hospitals than litigation.

## Competing interests

The authors declare that they have no competing interests.

## Authors’ contributions

All authors designed the study, analyzed the data, and drafted the manuscript. ER collected the data. All authors read and approved the final manuscript.

## Pre-publication history

The pre-publication history for this paper can be accessed here:

http://www.biomedcentral.com/1472-6963/14/38/prepub
